# Radiofrequency ablation of lung tumours

**DOI:** 10.2349/biij.2.3.e39

**Published:** 2006-07-01

**Authors:** PYT Goh

**Affiliations:** Department of Radiology, Mount Elizabeth Hospital, Singapore

**Keywords:** Radiofrequency ablation, lung tumours

## Abstract

Radiofrequency ablation (RFA) is a well-established local therapy for hepatic malignancies. It is rapidly emerging as an effective treatment modality for small lesions elsewhere in the body, in particular, the kidney and the lung. It is a relatively safe and minimally invasive treatment for small lung malignancies, both primary and secondary. In particular, it is the preferred form of treatment for non-surgical candidates.

This paper describes the technique employed for radiofrequency ablation of lung tumours, as well as the protocol established, at the Mount Elizabeth Hospital, Singapore.

## TECHNIQUE

### Mode of imaging

All cases are performed under computed tomography (CT) fluoroscopic guidance.

### RF system used

The Valley Lab Cool-tip radio frequency (RF) system (Valley Lab, Boulder, CO), with the 17-gauge single prong electrode, connected to a 200 W radiofrequency generator was used in all cases. The length of the exposed tip of the electrode chosen is 1 to 3 cm depending on the size of the lesion. In general, the length of the exposed tip should be greater than the diameter of the lesion. For lesions larger than 3 cm in diameter, multiple cycles of ablation are performed with re-positioning of the electrode between each cycle, to achieve overlapping areas of ablation larger than the lesion itself. The Cool-tip cluster electrode system was not used in any of these cases. Each cycle of ablation is 12 minutes, as per the standard protocol determined by the Valley Lab system for RF ablation under impedance control. Track ablation is performed at the end of the ablation cycle by switching off the cooling system after 11 minutes of ablation. The electrode is withdrawn when the temperature increases beyond 70ºC.

The other RF system available in our department is the LeVeen needle (Boston Scientific, Watertown, MA). This system was not used in any of our lung lesions. It was felt that the larger gauge needle and as well as its co-access or co-axial system subjected the patients to a higher risk of pneumothorax. Track ablation is not as easily achieved as in the Valley Lab system. The track is sequentially ablated at a power of 10 W and withdrawn following roll-off.

Furthermore, from a purely subjective point of view, this author finds the Cool-tip electrode a much easier electrode to place into a lesion accurately. It is a slimmer and a more flexible electrode. The technique of placing the electrode is to skewer the lesion so that the tip of the electrode is just distal to the far margin of the lesion. The stiffer and larger gauge co-access needle of the LeVeen system is more difficult to target lesions with, especially lesions with locations that require angling the needle superior or inferior to the skin entry point. The system also requires that the tip of the needle is a short distance away from the far margin of the lesion so that the tines of the LeVeen needle, when extended, achieve optimal coverage of the lesion. This takes some practice and experience and it may involve greater CT-fluoroscopy time. Furthermore, it is this author’s opinion that co-access or co-axial systems in the lung run a higher risk of pneumothorax.

### Pre-procedural evaluation

**Figure 1:**
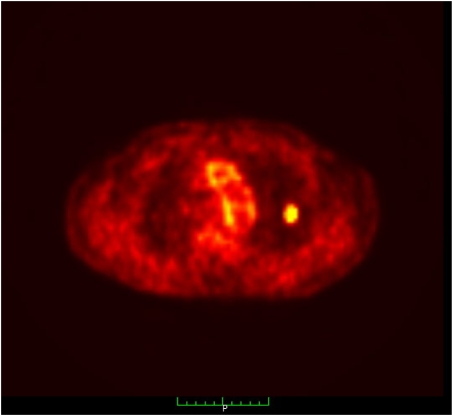
PET/CT image of single lung metastasis for RFA. This is a typical lesion and ideal for RFA.

**Figure 2:**
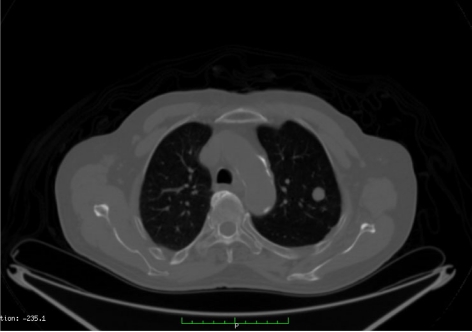
CT image of single lung metastasis pre-RFA.

The CT images are reviewed prior to ablation (Figures 1 and 2). The CT images should be recent; no more than four weeks prior to the procedure. The number, size, and location of the lesions are evaluated. Lesions close to vital structures need to be studied carefully as these may potentially be damaged by the heat generated during RFA. These include the heart, the main pulmonary vessels, the trachea, and the main bronchi. In general, the major vessels as well as the heart are protected from the heat as a result of the blood flow resulting in a heat sink effect [10]. Nevertheless, care is always taken to ensure that the electrode does not cross or abut a major vessel or the heart directly. Arbitrarily, the electrode is placed at a minimum of approximately 1 cm from these structures. Although the heat sink effect protects these structures from heat damage, it also implies that the portion of the lesion abutting these structures may also be protected form heat damage, resulting in sub-total ablation. In such cases, alcohol ablation of the portion of the tumour abutting the major vessels may be considered if RFA alone does not achieve complete ablation.

The clinical history of the patient is also reviewed. Care is taken to evaluate co-morbid conditions, especially other lung diseases, such as, chronic obstructive airway disease, bronchiectasis, and asthma. These cases have a higher risk as they will not tolerate complications of RFA of the lung, such as, pneumothorax and pulmonary haemorrhage. The theoretical risks of performing RFA on patients with pacemakers seem to be unfounded [10], and the procedure has been performed in these patients without significant complications. Nevertheless, a cardiologist, in particular, an electro-physiologist if available, should evaluate all patients with pacemakers. If the patient does not require the pacemaker constantly, it may be temporarily deactivated at the time of ablation, with external pacing on standby. If the patient is entirely dependent on the pacemaker and any disruption of its function cannot be tolerated, it may be prudent to explore other means of local ablative therapy, if available. These include laser ablation or microwave ablation, which do not employ RF energy.

The coagulation profile of the patient should always be assessed prior to the procedure.

Contraindications to RFA of lung lesions:

1. Coagulopathy
2. Extensive lung involvement with life expectancy of less than six months
3. Invasion of pericardium and mediastinum

### The procedure

The procedure is performed in the CT fluoroscopy room. A pre-RF, unenhanced CT of the thorax is performed. The site of the lesion and the entry point are chosen. The patient may be turned and re-positioned accordingly, e.g., in the prone or oblique position. The shortest possible path to the lesion, avoiding major structures and fissures, is usually selected.

A pleural drainage set is placed in the procedure room for use in the case of significant pneumothorax requiring immediate chest tube insertion. Usually, a simple 6Fr multi-purpose pigtail drainage catheter is all that is necessary.

As in all cases of radiofrequency ablation in our department, lung ablations are performed under heavy conscious sedation with the aid of an anaesthetist. Midazolam, fentanyl, and propofol are titrated during the procedure. The patient is placed on intra-nasal oxygen, and the vital signs are monitored. No intubation or general anaesthetic is required [8].

The 17-gauge RF electrode is inserted into the lesion under CT fluoroscopic guidance, under strict aseptic conditions.

**Figure 3:**
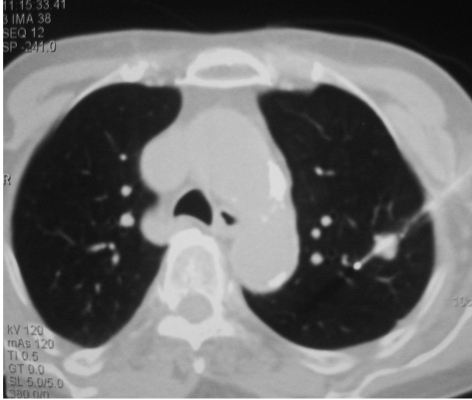
RF electrode inserted into lesion under CT fluoroscopic guidance. Note that the tip of the electrode has gone beyond the far margin of the tumour nodule to ensure that a good rim of normal lung tissue is also ablated.

After the tip of the electrode is successfully placed within the lesion (Figure 3), the portion of the electrode outside the patient’s body is supported by hand throughout the ablation cycle. This is necessary because the lung parenchyma is not as firm as the liver parenchyma. The weight of the electrode may cause it to change position, if left unsupported. Furthermore, respiratory movement, progressive atelectasis, and change in consistency of the lung parenchyma adjacent to the lesion during the ablation may result in a change in position of the electrode relative to the lesion. Therefore, intermittent CT fluoroscopy is performed during the ablation cycle, and the electrode position is readjusted appropriately. Arbitrarily, the lesion is imaged every three minutes during ablation.

The water-cooling system is switched off towards the end of the cycle, usually after 11 minutes of ablation, and the electrode is allowed to heat up. The electrode is withdrawn, after the temperature exceeds about 70ºC, to coagulate the track. The rationale behind track coagulation is to reduce the risk of tumour track seeding [9] and to reduce the risk of pneumothorax.

If the lesion is larger than 3 cm in diameter, the water-cooling system is left on for the full 12 minutes. The electrode is partially withdrawn at the end of the cycle and re-positioned for further ablation. The track is not coagulated at this point in time. Re-positioning and repeat ablation is performed up to three to four times, depending on the size of the lesion. If the lesion requires more than three to four cycles of ablation, the procedure will be re-scheduled for further ablation one month later. Track ablation is only performed during the final complete withdrawal of the electrode.

At present, there is no means of evaluating the adequacy of ablation of a lesion during the procedure. The end-point of ablation for small lesions (2 to 3 cm) is taken as the arbitrary 12-minute cut-off point in the Valley Lab Cool-tip system. The Cool-tip system set on impedance control will alternate between “on” (active) and “off” (inactive) modes depending on the impedance of the lesion being ablated. Long periods of inactivity (30 to 40 s) by the machine followed by short periods of activity (5 to 7 s) with impedance levels of greater than 100 ohms, indicate that the target volume of tumour has been ablated [7].

At our centre, the only other RFA system available is the LeVeen needle by the Boston Scientific Corporation. The end-point of ablation using this system is the roll-off when impedance increases rapidly following tumour cell death. Till date, however, we have not used the LeVeen system for RFA of lung lesions.

**Figure 4:**
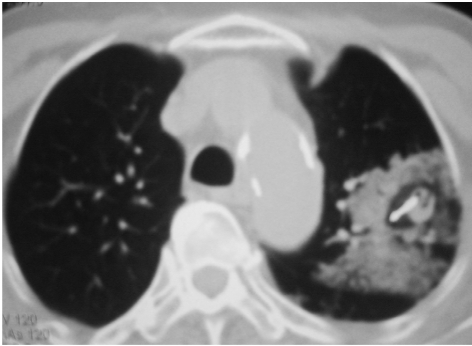
RFA in progress. Surrounding area of ground glass opacification is typical during ablation. There is also surrounding focal parenchymal haemorrhage, which is sometimes seen during RFA.

It has been suggested that an indication of complete tumour ablation is the presence of a good surrounding rim of ground glass opacification around the tumour (Figure 4). This is a useful sign to look out for when ablating lesions larger than 3 cm, which require multiple ablations and re-positioning of the electrode to achieve overlapping zones of ablation [10].

If a pneumothorax is sustained during the insertion of the electrode, prior to the actual placement of the electrode within the lesion, aspiration may be required. This is because the presence of a pneumothorax sometimes makes accurate placement of the electrode within the lesion difficult. A 6 Fr multiple side-hole drainage catheter is usually used. After complete aspiration of the pneumothorax, the catheter is left in-situ and closed off. If the pneumothorax, however, does not interfere with the placement of the electrode and if the patient is not clinically compromised, no aspiration is necessary.

An arbitrary maximum of 3 to 4 cycles of ablation are performed during a single session of RF ablation. This is irrespective of the number of lesions. If more cycles of ablation are required or if there are multiple lesions, further RFA is performed at a later date, usually at one-month intervals.

RFA for bilateral lung lesions during a single session is a relative contraindication. In such patients, it is better to perform RF for one side first and to schedule the lesions on the contra lateral side to be ablated, a month later. Depending on the clinical status of the patient and in the absence of complications during the ablation on one side, however, ablation of the contra lateral lesions may be performed during the same session.

### Post-procedure

Follow-up serial chest radiographs are performed after the procedure, as per standard post-lung biopsy protocol, to check for a pneumothorax or to track an existing one. Not all cases with pneumothorax require chest tube insertion. This is only inserted if the patient is symptomatic or if the pneumothorax is large.

The use of prophylactic antibiotics is debatable. At our centre, a single dose of peri-procedural broad-spectrum antibiotics is usually given.

Post-procedural analgesia is prescribed only if the patients require this. For subpleural lesions, there may be pain following the ablation and analgesia may be mandatory. Some patients with sub pleural lesions may develop a pleural effusion. This is drained percutaneuosly only if the patient is symptomatic.

**Figure 5:**
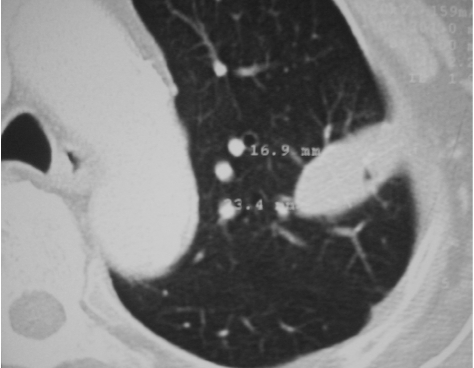
CT scan performed one-month post-RFA. This is a typical appearance of an oval area of coagulation necrosis scarring following RFA. Note that the area of scarring is larger than the original lesion, indicating a positive outcome.

Follow-up CT of the thorax (Figure 5) with intravenous contrast enhancement is performed at one month, three months, and six months post-procedure. After that, follow-up is at six-month intervals for two years and, then, yearly. If the ablation scar shows any evidence of increase in size or enhancement, repeat ablation may be performed. A review article by Rose *et al* [10] on radiofrequency ablation of lung cancer suggests that if there is an increase of at least 10 HU between the non-contrast scan and the contrast-enhanced scan, this is suggestive of residual viable tumour. Repeat ablation is then advised.

PET/CT scans are far more sensitive than contrast enhanced CT scans in detecting recurrent or residual viable tumour. Not all patients, however, can afford to be followed up with PET/CT. Where possible, a PET/CT is recommended at one month post-RFA and at one year, with contrast enhanced CT scans in between, at the intervals suggested above.

Tumour markers should be monitored if these were elevated, prior to RFA.
